# Editorial: Advances in genetics and molecular diagnosis in colorectal cancer

**DOI:** 10.3389/fonc.2023.1279195

**Published:** 2023-09-19

**Authors:** Farhadul Islam, Jorge Melendez-Zajgla

**Affiliations:** ^1^ Department of Biochemistry and Molecular Biology, University of Rajshahi, Rajshahi, Bangladesh; ^2^ Direccion General, Instituto Nacional de Medicina Genomica, Ciudad de Mexico, Mexico

**Keywords:** colorectal < cancer type, editorial, topic, cancer, advances

Colorectal cancer (CRC) is the third most common tumor worldwide. Paradoxically, while the global incidence of CRC has been declining over the last decade, cases among patients younger than 55 years have increased from 11% in 1995 to 20% in 2019. Additionally, CRC localization has shifted from the right to the left colon, and unfortunately, it is being diagnosed at later stages. The genomic basis for CRC progression was one of the first to be elucidated. However, our understanding of the molecular heterogeneity in these tumors has been lacking. Thanks to large cancer genomic initiatives such as the TCGA (1) and ICGC (2), most of the somatic mutations in these tumors have been discovered. RNA sequencing of large cohorts of CRC neoplasia has also provided important information about the expression cassettes that drive progression (Zhong et al.). These datasets have been instrumental in exploring new questions, such as new molecular classifications (Lu et al.), the role of microenvironment (Jun et al), including the immune-tumor response (Yuan et al.), and the recent use of expression signatures as prognostic tools. These advancements are offering valuable insights into the nature of CRC and paving the way for improved diagnoses and treatments.

The present Research Topic presents several interesting articles that contribute to these areas. For example, Nguyen et al. took advantage of the known mutated genes in CRC and used a gene panel assay to sequence tumors and create a circulating-free DNA (cfDNA) personalized test that was able more sensitive than carcinoembryonic antigen in detecting neoplasia. This “in house” test could help low-to-medium income countries to provide a surveillance method in CRC or even help to characterize possible actionable mutations.

The extensive genomic characterization of CRC has enabled researchers to search for gene expression signatures that have prognostic and predictive potential. This is done with the goal of stratifying patients. The field is rapidly evolving, as it could lead to a significant shift in cancer treatment approaches. However, currently, only a small number of signatures, primarily in breast cancer, have demonstrated their clinical implications. Jun et al. provide evidence that a novel molecular subtype (TMERSS), based on a tumor microenvironment signature has not only higher antitumor immune cells number, but also is associated with a better prognosis in patients, associated with a better response to Cetuximab and immunotherapy. Similarly, Ma et al. report a N6-methyladenosine-related gene prognostic index (m6A-GPI) that is associated with a shorter disease-free survival and notable differences in a diverse array of tumor variables, such as copy number alterations and homologous recombination defects.

One of the most intriguing reports in the Topic is the Qin et al. findings that show that benign gallbladder disease was positively correlated with the presence of CRC, especially of the right side. For several years, a controversial association of gallbladder disease and CRC has been proposed. This could be consistent with the known common risk factors for both diseases, such as obesity, smoking, low-fiber diet, etc. A plausible mechanism could be the alterations in bile flow, with would increase inflammation, a known CRC risk factor. In their report, Qin et al. analyzed 7160 CRC cases and showed that patients with gallbladder disease had a higher risk for colon cancer than rectal cancer (20.4% vs 18.2%, p =0.024). These results need to be replicated in other oncologic centers, preferably in additional countries, and a more granular data analysis would help to define new or specific variable associations.

The articles presented in this topic provide a glimpse of the current research trends in CRC. It is noteworthy that most of them are based on the large cancer initiatives that elucidated the mutational landscape of these tumors. Beside generating new hypothesis and a deep understanding of CRC progression, these articles should eventually lead to new diagnostic, prognostic, and therapeutic approaches.

## Author contributions

JM-Z: Conceptualization, Writing – original draft, Writing – review & editing. FI: Writing – review & editing.
